# Prostate minimally invasive procedures: complications and normal vs. abnormal findings on multiparametric magnetic resonance imaging (mpMRI)

**DOI:** 10.1007/s00261-021-03097-6

**Published:** 2021-05-11

**Authors:** Thanh-Lan Bui, Justin Glavis-Bloom, Chantal Chahine, Raj Mehta, Taylor Wolfe, Param Bhatter, Mark Rupasinghe, Joseph Carbone, Masoom A. Haider, Francesco Giganti, Simone Giona, Aytekin Oto, Grace Lee, Roozbeh Houshyar

**Affiliations:** 1 Department of Radiological Sciences, University of California, Irvine, Orange, CA 92868-3201 USA; 2 Joint Department of Medical Imaging, Sinai Health System, University Health Network, University of Toronto, Toronto, ON Canada; 3 Department of Radiology, University College London Hospital NHS Foundation Trust, London, UK; 4 Division of Surgery & Interventional Science, University College London, London, UK; 5 Department of Urology, Frimley Park Hospital, Frimley, Camberley UK; 6 Department of Radiology, University of Chicago, Chicago, IL USA; 7 Department of Radiological Sciences, University of California, Irvine, Bldg. 1 Route 140, Orange, CA 92868-3201 USA

**Keywords:** Irreversible electroporation, Photodynamic therapy, High-intensity focused ultrasound, Focal cryotherapy, Focal laser ablation, Prostatic artery embolization, Prostatic urethral lift procedure, Benign prostatic hyperplasia, Prostate cancer

## Abstract

Minimally invasive alternatives to traditional prostate surgery are increasingly utilized to treat benign prostatic hyperplasia and localized prostate cancer in select patients. Advantages of these treatments over prostatectomy include lower risk of complication, shorter length of hospital stay, and a more favorable safety profile. Multiparametric magnetic resonance imaging (mpMRI) has become a widely accepted imaging modality for evaluation of the prostate gland and provides both anatomical and functional information. As prostate mpMRI and minimally invasive prostate procedure volumes increase, it is important for radiologists to be familiar with normal post-procedure imaging findings and potential complications. This paper reviews the indications, procedural concepts, common post-procedure imaging findings, and potential complications of prostatic artery embolization, prostatic urethral lift, irreversible electroporation, photodynamic therapy, high-intensity focused ultrasound, focal cryotherapy, and focal laser ablation.

## Introduction

Prostate cancer is one of the most common cancers in the United States [1]. Although roughly 1 in 9 men are diagnosed with prostate cancer in their lifetime, the majority of cases are indolent and associated with a high 5-year survival. Accurate differentiation of nonaggressive versus aggressive cancer types is essential to minimizing risks of overtreatment, particularly in elderly patients with comorbidities who may be poor surgical candidates [2].

Another highly prevalent disease of the prostate is benign prostatic hyperplasia (BPH). It affects most men within their lifetime, with a prevalence of 90% by the ninth decade of life [3]. As the prostate enlarges, it compresses the prostatic urethra, resulting in difficulties with urination termed “lower urinary tract symptoms” (LUTS) and complications including incontinence, urinary retention, and urinary tract infections. Lifestyle modifications and pharmacologic treatments are typically employed as first-line therapy, with prostatectomy traditionally reserved for persistent, debilitating symptoms.

In recent years, a variety of less invasive alternatives to traditional prostate surgery have gained popularity. These minimally invasive procedures aim to mitigate the risks of traditional prostatectomy, which include damage to the neurovascular bundle and urethra and can result in sexual dysfunction and urinary incontinence [4]. These novel procedures include treatments for BPH and localized prostate cancer in select patients. Minimally invasive procedures for localized prostate cancer include irreversible electroporation, photodynamic therapy, high-intensity focused ultrasound, focal cryotherapy, and focal laser ablation; minimally invasive procedures for treating BPH include prostatic artery embolization and prostatic urethral lift.

To evaluate the prostate, multiparametric magnetic resonance imaging (mpMRI) is increasingly employed and consists of T1-weighted images (T1WI), T2-weighted images (T2WI), diffusion-weighted images (DWI), apparent diffusion coefficient maps (ADC), and dynamic contrast-enhanced images (DCE) [5]. It has high sensitivity, specificity, and negative predictive value in detecting prostate cancer [6–8]. Prostate mpMRI images are interpreted and reported using the Prostate Imaging-Reporting and Data System (PI-RADSv2), which evaluates lesions utilizing T2WI, DWI, and DCE and produces an overall risk assessment ranging from 1 to 5 [9]. The risk category can be used to determine which lesions should be biopsied and are likely to demonstrate primary clinically significant prostate cancer [10, 11]. While PI-RADSv2 is useful to detect primary clinically significant prostate cancer, it is not designed to plan treatment. Additionally, it is not appropriate in the post-treatment setting, as the criteria for the detection of recurrent prostate cancer after minimally invasive procedures are likely distinct from PI-RADSv2 criteria.

As minimally invasive procedure and prostate mpMRI volumes continue to increase, radiologists must be familiar with the common procedures, complications, and expected post-procedure appearance of the prostate. These procedures can distort the prostate appearance and may introduce MRI artifacts, which can increase the risk for misinterpretation. This paper offers a review of contemporary minimally invasive prostate procedures for BPH and localized prostate cancer, associated complications, and normal post-procedure imaging findings.

### Minimally invasive procedures for BPH

#### Prostatic artery embolization

##### Background

Prostatic artery embolization (PAE) was approved by the United States Food and Drug Administration (FDA) in 2017 for the treatment of LUTS secondary to BPH [12]. It is currently indicated for patients with special risks for surgery, sexually active men who wish to avoid the risk of retrograde ejaculation, patients with a prostate volume greater than 65 mL, those with recurrent bleeding secondary to BPH, or patients with a permanent bladder catheter.

##### Procedure

Under local anesthesia and moderate sedation, arterial access is achieved to the common femoral artery [12]. After evaluation of the iliac and prostatic arterial anatomy by angiography and often with confirmation of prostate perfusion by computed tomography (CT), the angiographic endpoint for embolization is determined. Embolization of the prostatic artery utilizing 100–300 and/or 300–500 μm microsphere particles causes ischemic necrosis in the prostate, leading to a reduction in prostate size and LUTS.

##### Post-procedure imaging (Fig. 1)

After PAE, infarcts on the embolized side(s) are characterized by initial hyperintensity on T1WI, which is greatest 1–3 months after the procedure, and hypointensity on T2WI [13, 14]. Over time, these infarcts decrease in size and become isointense on both sequences. They are typically not seen on these sequences at the 12-month follow-up. On DWI, the infarcts demonstrate decreased signal intensity starting at 1-month post procedure. This decrease in signal intensity corresponds with prostatic softening and is even more evident at 12 months post procedure, which is seen as a sign of treatment success [14]. Twelve months is also when a statistically significant decrease in prostate volumes is typically seen, with volumes decreasing approximately 20–40% [13–15].

**Fig. 1:**
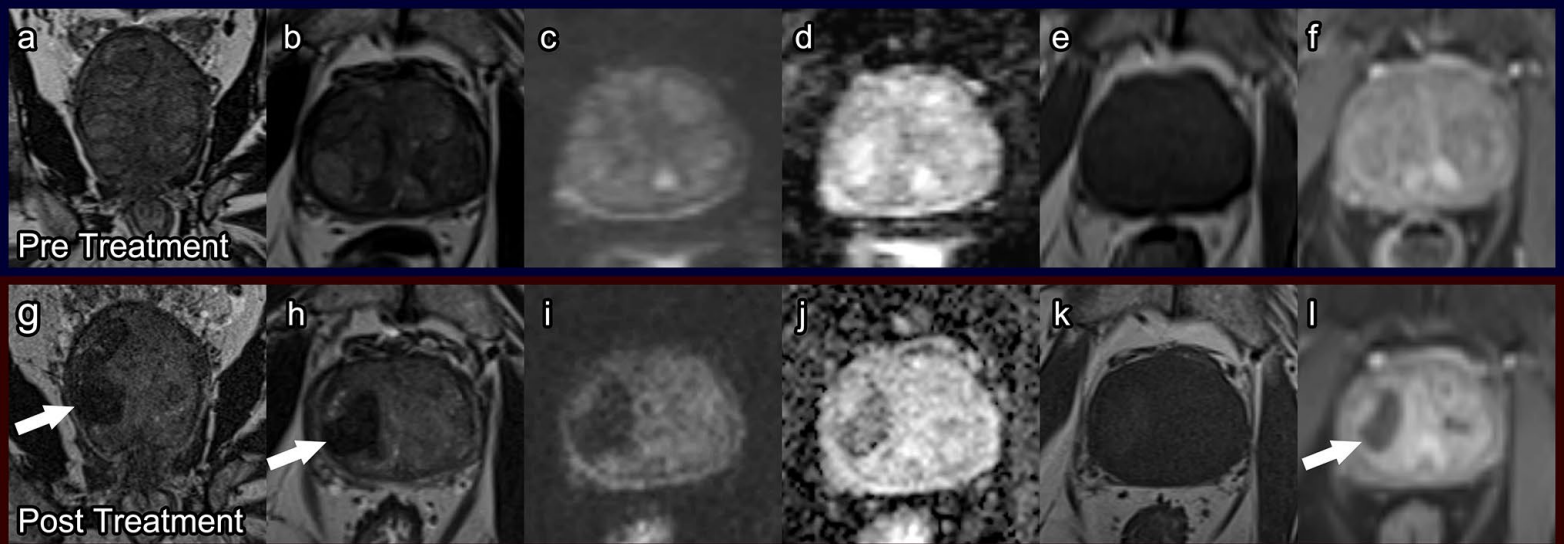
Prostatic artery embolization. Pre-treatment prostate MRI: Coronal T2 (**a**), axial T2 (**b**), axial DWI (**c**), ADC (**d**), pre-contrast T1 (**e**), and post-contrast T1 (**f**) demonstrate prostatomegaly with findings of benign prostatic hyperplasia, without diffusion restriction and with normal enhancement. Post-PAE prostate MRI at 4 months: Coronal T2 (**g**), axial T2 (**h**), axial DWI (**i**), ADC (**j**), pre-contrast T1 (**k**), and post-contrast T1 (**l**) demonstrate T2 dark areas of chronic ischemia with hemosiderin deposition (arrows) no significant diffusion restriction (**i**), and new hypoenhancement associated with ischemic tissue (arrow, **l**)

While pre- and post-procedure mpMRI are part of many PAE protocols, there is no consensus on follow-up imaging intervals for monitoring clinical success. Some studies have suggested repeating mpMRI as soon as 1-month post procedure, while others recommend imaging to begin at the 3-month follow-up [13, 15]. Additionally, some studies recommend mpMRI at multiple time points, while others believe that a one-time post-procedure mpMRI demonstrating the above changes is adequate. Of note, some clinicians opt to use ultrasound only post-PAE to measure the decrease in prostate volume. Additionally, in the absence of post-procedure symptoms, some clinicians might argue that post-procedure imaging is unnecessary.

##### Complications

Major complication rates for PAE are reported to be between 0.1 and 0.4% [16]. The most common complication of PAE is cystic transformation within an area of infarct, which is seen as high signal intensity on T2WI and low signal intensity on T1WI, typically at 12 months post procedure [17]. Another common complication, usually seen within 72 h of PAE, is “post-embolization syndrome” with symptoms including pain, nausea, vomiting, mild fever, and dysuria [15]. Patients may also experience bladder ischemia, sometimes requiring additional surgery, and persistent perineal pain. Minor complications include dysuria, acute urinary retention, hematospermia, minimal rectal bleeding, and urinary tract infections.

#### Prostatic urethral lift

##### Background

The prostatic urethral lift procedure, commonly performed with a UroLift^®^ device (NeoTract-Teleflex, Pleasanton, CA, USA), was first introduced as a feasible treatment for lateral lobe enlargement BPH in 2011 and received FDA approval in 2013 [18]. Because it has reduced rates of procedure-related sexual dysfunction and shorter length of hospital stay, it is an ideal option for men who are sexually active; those who do not want to take lifelong medication; men who have failed medical management; or those who do not want cavitating surgery. Patient eligibility depends on prostate volume and median lobe size [19, 20]. If the median lobe is too large and obstructing, a lateral lobe prostatic urethral lift procedure is contraindicated.

##### Procedure

The prostatic urethral lift device is delivered under cystoscopic guidance and placed at least 1.5 cm distal to the bladder neck, near the anterolateral prostate area [18, 19]. It must be angled such that when the needle is deployed, its path will be parallel to the bladder neck. Once in place, the device is deployed and delivers a prosthesis that retracts the lateral lobes of the prostate, which in turn increases flow through the urethra. Flow is assessed cystoscopically, and additional devices can be implanted anterior to the verumontanum as needed to keep the urethra open. After all the devices are deployed and adequate flow is confirmed cystoscopically, the procedure is complete.

##### Post-procedure imaging (Fig. 2)

**Fig. 2:**
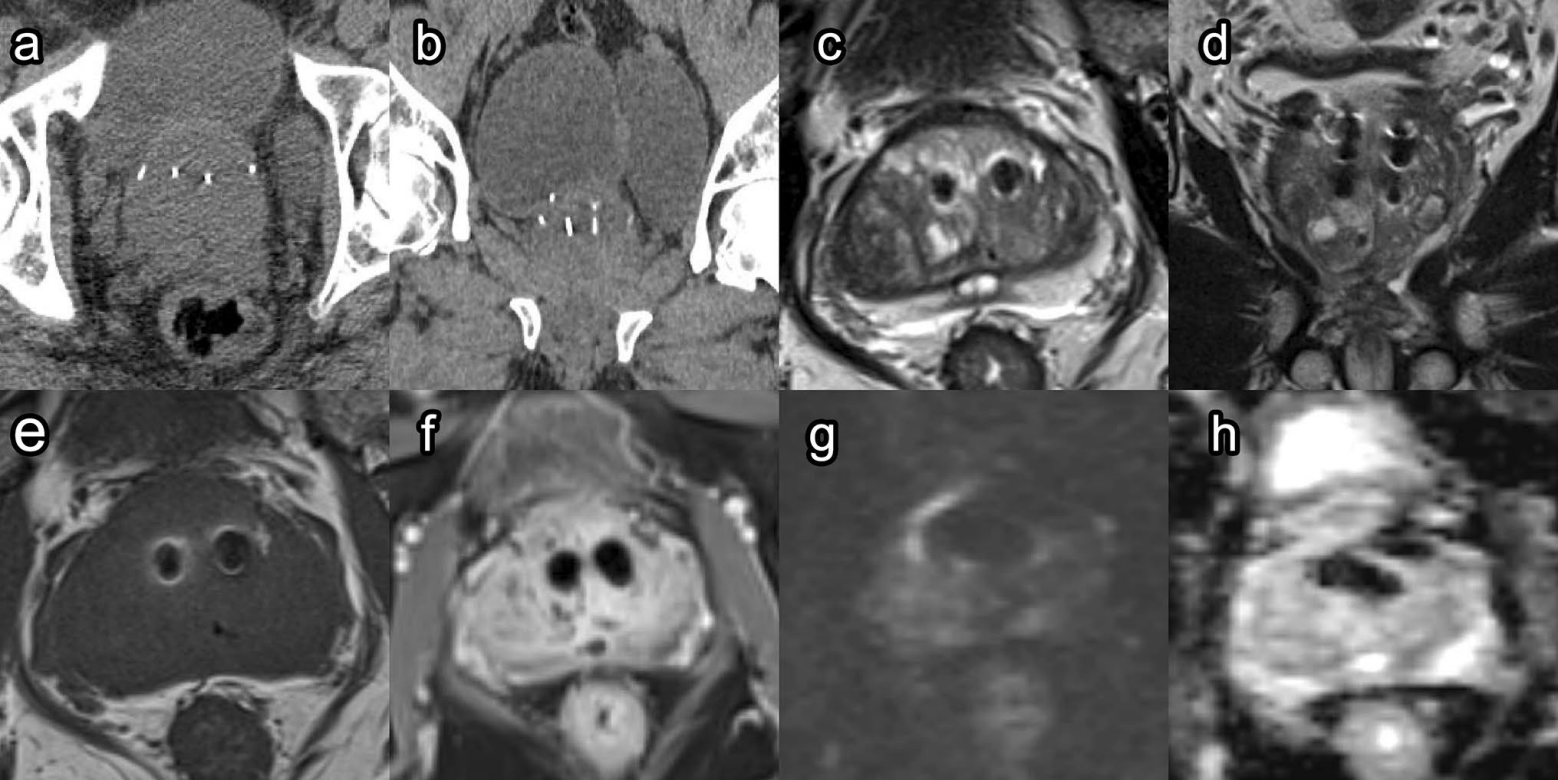
Prostatic urethral lift. Post-prostatic urethral lift prostate CT: Axial (**a**) and coronal (**b**) images of a patient with Urolift® implant. Metallic attenuation objects are noted in appropriate position along the bilateral median lobes. Post-prostatic urethral lift prostate MRI: Axial T2 (**c**), coronal T2 (**d**), pre-contrast T1 (**e**), and post-contrast T1 (**f**) images demonstrate a metal susceptibility artifact causing a signal void with peripheral hyperintensity in the region of the implant. DWI at B1400 (**g**) and ADC (**h**) images demonstrate exaggerated signal void in the region of the implant

The prostatic urethral lift prosthesis that is implanted into the patient is composed of a stainless-steel tab and a nitinol tab, which are bridged by suture [21]. The nitinol portion does not show up on imaging. However, the stainless-steel urethral part of the prostatic urethral lift device can generate a 10–15 mm susceptibility artifact with peripheral hyperintensity. This artifact becomes more prominent on DWI and ADC images and can obscure evaluation of the transition zone, and in some cases the peripheral base (Fig. 2).

Obscurement of the transition zone can make interpretation of imaging for prostate cancer more challenging [22]. Therefore, prior to prostatic urethral lift, patients should be made aware of this limitation, especially if they are high risk for prostate cancer. Additionally, if there is any evidence suggesting that prostate cancer may be developing in a patient who has had a prostatic urethral lift, it is essential for clinicians to include the prostate anterior zone during ultrasound-guided biopsies [21].

##### Complications

Overall complication rates of prostatic urethral lift are low [19, 20]. The most reported adverse events include dysuria, hematuria, urgency, and pelvic pain. However, most of these complications are mild and self-limiting.

### Minimally invasive procedures for localized prostate cancer

Minimally invasive procedures for treating localized prostate cancer include high-intensity focused ultrasound (HIFU), cryotherapy, irreversible electroporation (IRE), focal laser ablation (FLA), and photodynamic therapy (PDT). These procedures are highly effective and all eventually lead to fibrosis of the treatment area. As such, on delayed mpMRI imaging, post-treatment prostates are expected to have a similar appearance irrespective of the procedure performed—the treated area of the prostate will have T2 dark signal [23–27]. However, short-term post-treatment imaging changes vary based on the procedure.

#### High-intensity focused ultrasound

##### Background

HIFU was first introduced as a minimally invasive treatment for prostate cancer in the 1990s and became FDA approved for prostate tissue destruction in 2015 [28]. The procedure is performed for patients with low- and intermediate-risk localized prostate cancer; patients requiring salvage therapy for recurrent prostate cancer after radical prostatectomy, hormone ablation, or radiation therapy; patients with advanced prostate cancer for neoadjuvant debulking; and patients with castration-resistant prostate cancer [29].

##### Procedure

Under general or spinal anesthesia, a HIFU probe is inserted transrectally [28]. The target lesion(s) are visualized and mapped with ultrasound, often utilizing preprocedural MRI fusion guidance. A series of pulsed high-energy sound waves are delivered through the probe to the targeted area, which rapidly raises the target tissue’s temperature and promotes cell death. The probe is then repositioned, and the procedure is repeated at other target sites as needed. HIFU can be performed using ultrasound targeting or MRI in-bore targeting.

##### Post-procedure imaging (Fig. 3)

**Fig. 3:**
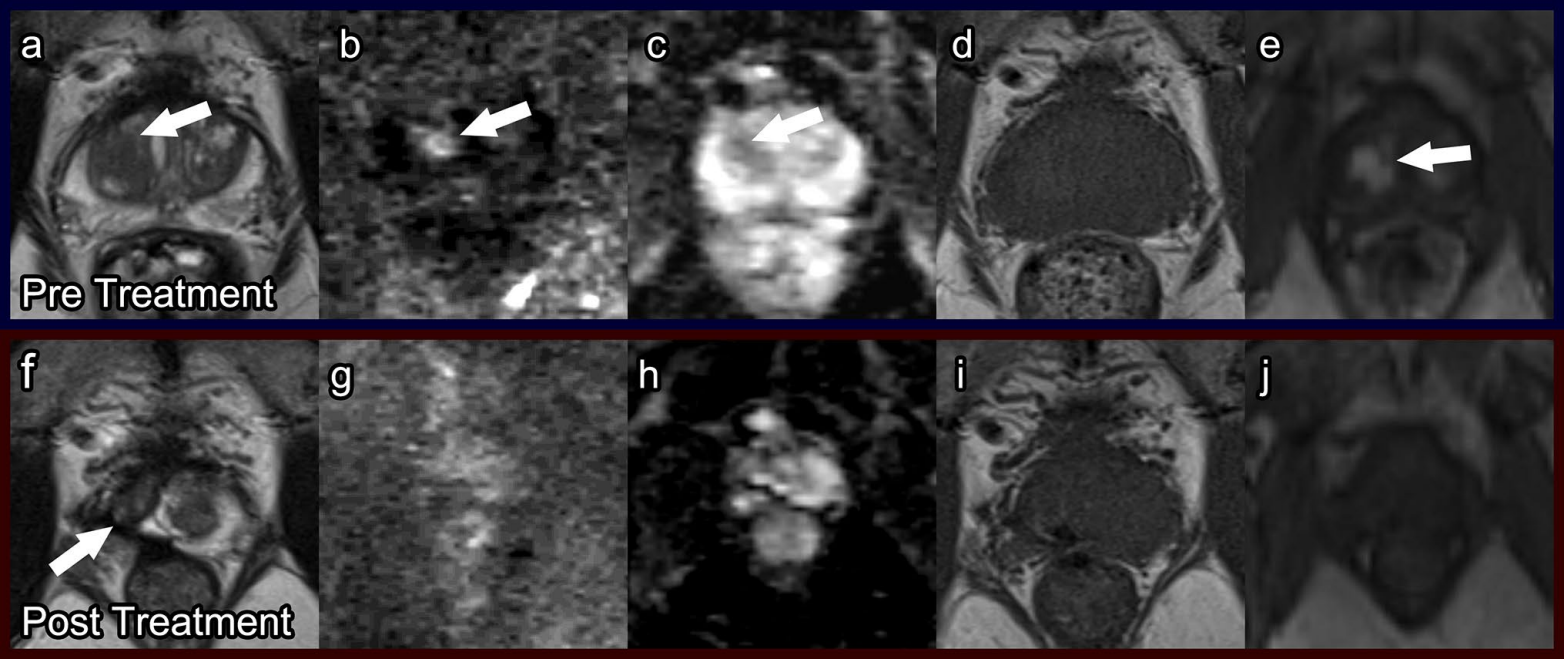
High-intensity focused ultrasound. Pre-treatment prostate MRI: Axial T2 (**a**), DWI (**b**), ADC (**c**), pre-contrast T1 (**d**), and post-contrast T1 (**e**) demonstrate a PI-RADS 5 lesion (arrows) in the right mid-transition zone. Biopsied as Gleason score 4 + 3 prostate cancer. Post-HIFU prostate MRI at 1 year: Axial T2 (**f**), DWI (**g**), ADC (**h**), pre-contrast T1 (**i**), and post-contrast T1 (**j**) demonstrate linear T2 dark scarring (arrow) and prostatic atrophy without restricted diffusion or hyperenhancement

Immediately after HIFU, it is difficult to distinguish the peripheral and transition zones due to complete loss of zonal anatomy [30]. One to three months post procedure, a “double ring” sign (heterogeneous T2-hypointense treatment zone with a surrounding hyperintense ring) can be seen, which is unique to post-HIFU imaging [26]. The amount of enhancing tissue at this time has been found to correlate with prostate volume at 6 months, PSA level nadir, and biopsy evidence of residual cancer. Therefore, it can be an early indicator of treatment success. The “double ring” sign typically disappears after 6 months. At 6 months, the post-treatment prostate shows low T2 signal intensity with poorer definition of the prostate capsule (Fig. 3). Prostate volume by this time will have decreased 40–60%.

**Fig. 4:**
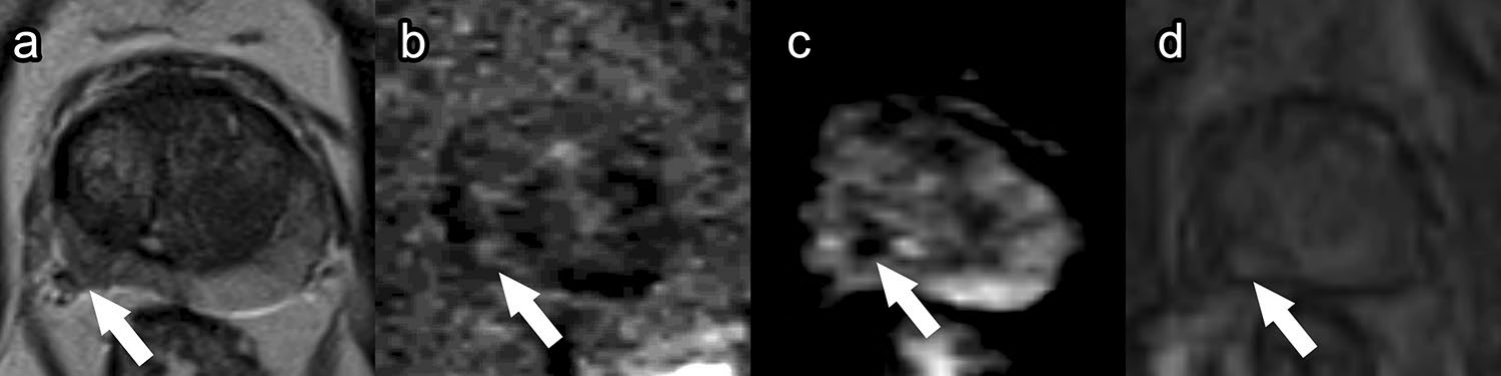
Recurrence in a patient after high-intensity focused ultrasound. Post-treatment prostate MRI: Axial T2 (**a**), DWI (**b**), ADC (**c**), and post contrast T1 (**d**) demonstrate a right posterolateral peripheral zone lesion with heterogeneous T2 dark signal, restricted diffusion and early arterial hyperenhancement (arrows). Biopsied as Gleason 3 + 4 recurrent prostate cancer with perineurial invasion

Formal timelines do not exist for follow-up mpMRI after HIFU. However, based on the expected post-procedure imaging findings, it has been proposed to obtain repeat mpMRI at 6–12 months [26]. Cancer recurrence can be evident as early as 6 months post treatment and is seen as a hypointense nodular lesion on T2WI with restricted diffusion on DWI and rapid wash-in and wash-out of contrast on DCE (Fig. 4) [31]. Cancer recurrence and fibrosis both have low signal on ADC and T2; however, recurrence demonstrates high signal on DWI and early enhancement while fibrosis may have late enhancement. DCE sequences are most useful in distinguishing recurrence from post-procedure fibrosis [32].

##### Complications

The most common complications of HIFU include prolonged voiding dysfunction, urinary retention, and erectile dysfunction [26]. The urethral wall, adjacent neurovascular bundle, rectal wall, and pelvic bone are vulnerable to thermal damage during HIFU. These structures may show hypoenhancement due to necrosis immediately after treatment but may partially or completely regain their enhancement at later follow-up. Urethrorectal fistula (< 1%) is a rare complication that typically self-resolves.

#### Cryotherapy

##### Background

Guided cryotherapy, also called cryoablation or cryosurgery, became a Medicare and Medicaid approved primary treatment for prostate cancer in 1999 [33]. Current guidelines recommend whole gland cryotherapy for low- and intermediate-risk patients who are poor candidates for prostatectomy or radiotherapy. When performed for curative purposes, whole gland cryotherapy should be done in patients with T1–T3 tumors without metastatic disease [34]. Prostate size is also a factor taken into consideration, as cryotherapy is more difficult to perform on large prostates (size greater than 60 g). Focal cryotherapy can also be done, but no current guidelines exist for its use.

##### Procedure

Under general anesthesia, cryoneedles are inserted into the prostate through either a transrectal or transperineal approach under ultrasound or MR guidance [35]. A urethral warming catheter and thermal sensor are positioned between the anterior rectal wall and prostate capsule to monitor and protect adjacent structures. The cryoneedles are then cooled with argon gas until the target area reaches a temperature of − 40 °C to − 80 °C. The target area is thawed and refrozen several times, causing cell death and promoting coagulative necrosis. The resultant formation of ice crystals and an ice ball distorts and destroys local tissue architecture.

##### Post-procedure imaging (Fig. 5)

**Fig. 5:**
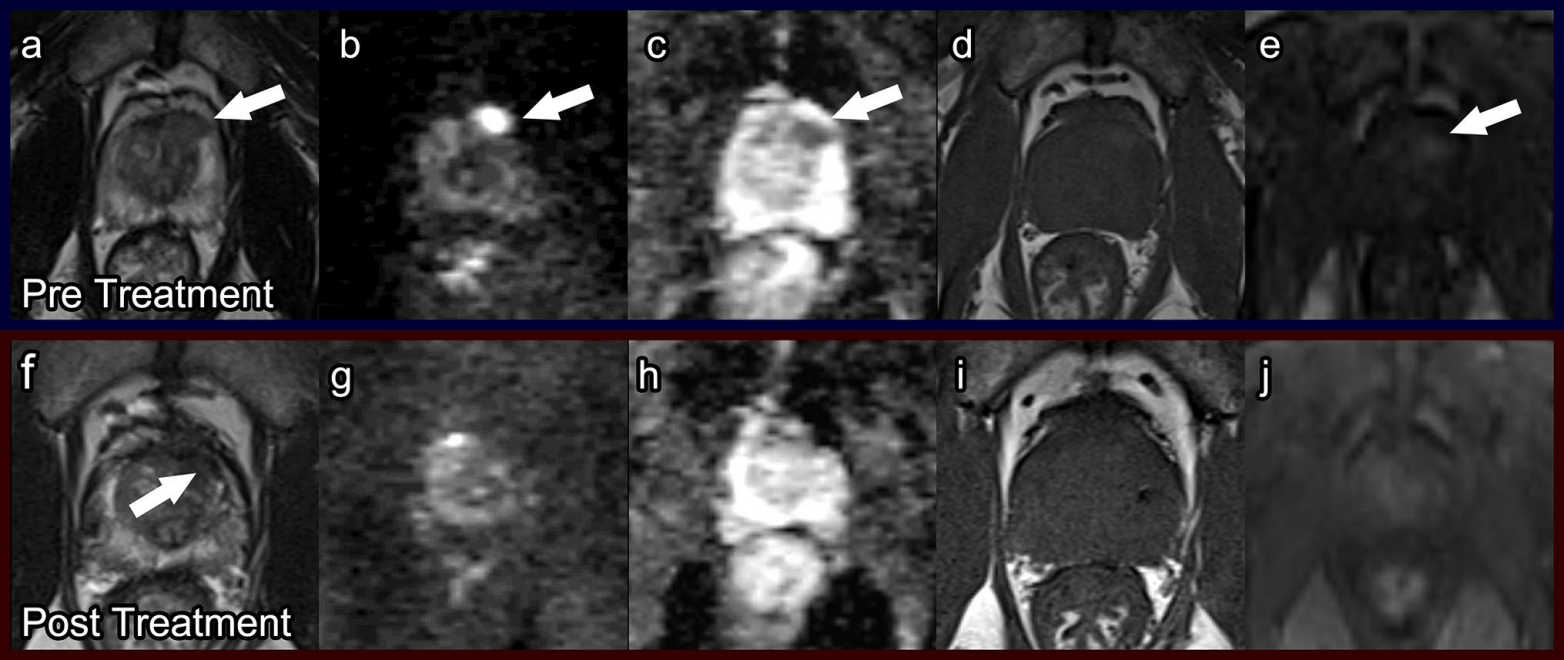
Cryotherapy. Pre-treatment prostate MRI: Axial T2 (**a**), DWI (**b**), ADC (**c**), pre-contrast T1 (**d**), and post-contrast T1 (**e**) demonstrate a PI-RADS 5 lesion (arrows) in the left mid-anterolateral peripheral zone. Biopsied as Gleason score 3 + 4 prostate cancer. Post-cryoablation prostate MRI at 1 year: Axial T2 (**f**), DWI (**g**), ADC (**h**), pre-contrast T1 (**i**), and post-contrast T1 (**j**) demonstrate ovoid T2 dark scarring (arrow), mild prostatic atrophy without restricted diffusion or hyperenhancement

In the time period closely following cryotherapy, imaging findings are distinctive due to the repetitive freezing and thawing of prostate tissue. During cryotherapy, the ice ball produces a strong T1 signal void with high-intensity rim [27]. After the procedure, DCE sequences consistently show an enhancing rim around a nonenhancing area. T1WI shows heterogeneous enhancement intermixed with areas of necrosis, and a diffusely hypointense T2 signal is observed within the peripheral zone with impeded diffusion on DWI (Fig. 5). Two months after treatment, thickening of the prostate capsule, rectal wall, and urethra can be seen along with a decrease in prostate volume [27]. When cryotherapy is used to destroy the whole prostate, imaging is often not useful after the procedure to detect recurrence [32]. Frequently, the damaged zone is too large, and any untreated prostate or cancerous tissue is small (less than 5 mm) and irregularly shaped, making it challenging to detect on mpMRI. Often times, cryotherapy is used for focal treatment, and the damaged gland is limited by laterality of treatment side. Post-treatment surveillance for local recurrence with mpMRI is best appreciated on DCE sequences [30].

##### Complications

Erectile dysfunction is almost an expected outcome after whole gland cryosurgery because the neurovasculature cannot be preserved [33]. This is especially common with posterior cryotherapy. Therefore, some clinicians perform cryotherapy on anterior lesions and opt for another procedure, such as HIFU, for posterior lesions. Other common complications include damage to the urethra, urinary incontinence, irritative urinary symptoms, and urinary obstruction. Urethrorectal fistula is an uncommon adverse event (< 1%) [35].

#### Irreversible electroporation

##### Background

IRE induces programmed cell death and apoptosis using very short and strong pulsed electric fields [36]. It gained FDA approval in 2019 and is performed in localized low- to intermediate-risk prostate cancer. It has been used for treatment of high-risk prostate cancers (mostly in Europe).

##### Procedure

Under general anesthesia, a transurethral urinary catheter is inserted [36]. Monopolar electrodes are then placed transperineally and positioned around target lesions using MRI-TRUS fusion technique. Correct electrode position and inter-electrode distances are confirmed. IRE pulses are emitted with frequencies appropriate to specific protocols.

##### Post-procedure imaging (Fig. 6)

**Fig. 6:**
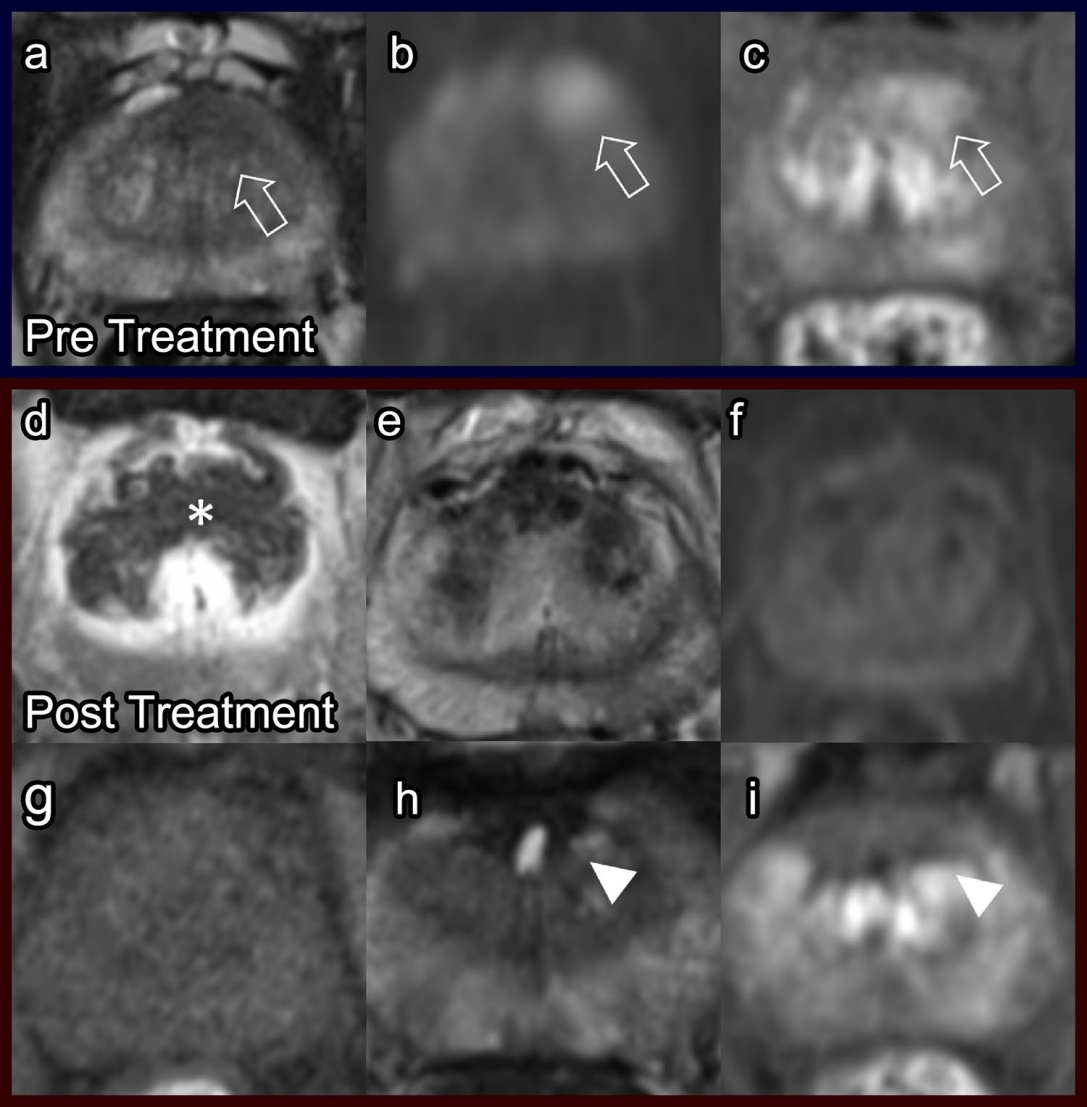
Irreversible electroporation. Pre-treatment prostate MRI: Axial T2 (**a**), DWI (**b**) and post-contrast T1 (**c**) demonstrate an anterior left mid-gland lesion (arrows). Biopsied as Gleason score 3 + 4 prostate cancer. Post-IRE prostate MRI at 7 days: DCE (**d**), T2 (**e**), DWI (**f**), and pre-contrast T1 (**g**) show a post-procedure necrotic area (asterisk) and heterogeneous decreased T2 signal. Post-IRE prostate MRI at 3 years: T2 (**h**) and DCE (**i**) indicate residual fibrosis (arrowheads), with no signs of recurrence at the treatment site

Immediately post IRE, the prostate volume increases significantly, which is thought to be due to necrosis [37]. At 24–72 h after the procedure, the ablation zone is heterogeneously or homogenously hypoattenuating with hyperattenuating margins on T1WI, possibly due to reactive hyperemia and/or sustained vascularization [38]. This appearance is characteristic of IRE. At 1 month, post-contrast sequences demonstrate areas of nonenhancement, and heterogeneous signal intensity is seen on T2WI [30]. Additionally, areas of low T2 signaling can be observed, which represent fibrosis (Fig. 6) [25]. By 6–12 months, the prostate volume has decreased considerably.

Studies suggest that post-procedure follow-up imaging after IRE should include a repeat mpMRI at 6–12 months [39]. Subsequent mpMRI can be obtained depending on patient-specific factors. Residual or recurrent disease demonstrates low T2 signal, asymmetrical enhancement on DCE, and high signaling on DWI with restricted diffusion on ADC [25]. DCE sequences are the most sensitive for detecting recurrence [30].

##### Complications

Complications are relatively uncommon with IRE, but the most common adverse events include urinary retention and dysuria [37]. They occur more frequently in patients with large prostates or when a large area of prostate was treated. Notably, IRE preserves collagenous and protein or lipid-based structures, such as vasculature, and ductal networks, such as the urethra. A large retrospective European study of 429 patients who underwent IRE reported rates of 0% for urinary incontinence and only 11.3% for erectile dysfunction [36].

#### Focal laser ablation (FLA)

##### Background

The use of FLA is still being investigated with ongoing studies, and no guidelines exist for its use [40]. Larger randomized control trials have yet to be conducted. FLA is currently recommended in select outpatients with low- to intermediate-risk prostate cancer with Gleason Score ≤ 4 + 3.

##### Procedure

Under direct MR guidance, a laser fiber is inserted either transperineally or transrectally through a grid towards the target lesion [24, 41]. Once the correct position is confirmed, a laser, typically potassium-titanyl-phosphate or holmium, is activated for 1 to 2 min using 6–25 W to heat the target lesion to temperatures as high as 60 °C. This thermally destroys the lesion. Temperature is usually monitored in the neurovascular bundle to help prevent neurovascular bundle damage. Repositioning occurs as needed to maximize treatment area. A repeat T2-weighted image is then re-obtained to confirm target lesion cooling. While FLA is typically performed in-bore, studies have demonstrated that it is safe and feasible in the clinic setting also [42].

##### Post-procedure imaging (Fig. 7)

**Fig. 7:**
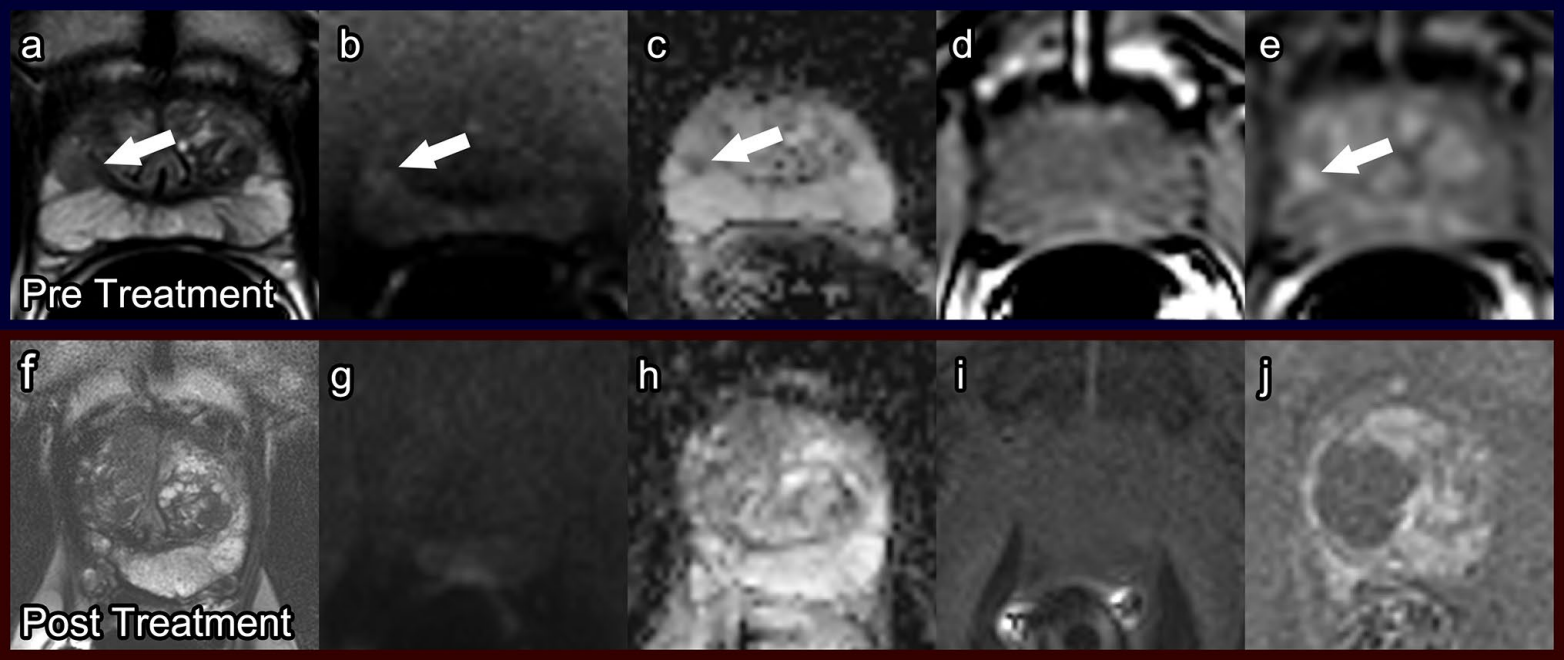
Focal laser ablation. Pre-treatment prostate MRI: Axial T2 (**a**), DWI (**b**), ADC (**c**), pre-contrast T1 (**d**), and post-contrast T1 (**e**) demonstrate a lesion at the right lateral mid-gland to apex peripheral zone (arrows). Post-focal laser ablation prostate MRI at 1 year: Axial T2 (**f**), DWI (**g**), ADC (**h**), pre-contrast T1 (**i**), and post-contrast T1 (**j**) demonstrate T2 hypointensity, atrophy, and retraction, with hypoenhancement in the ablated right lateral gland

The treatment area after FLA is better defined compared to after other focal therapies because of the targeted nature of the procedure. Immediately after FLA, post-contrast sequences typically show nonenhancing lesions, which decrease in size at 3 months post treatment and disappear by 1 year [24]. DWI shows restricted diffusion, which resolves by 6 months after FLA (Fig. 7) [30]. T2WI demonstrates patchy, low signal intensity within the treatment area at 3-month follow-up, which is especially apparent by 1 year [24].

There is no standard time interval for repeat mpMRI after FLA. However, one study suggested at least one repeat mpMRI 6–12 months post procedure with periodic serial mpMRI performed after depending on patient characteristics [39]. Others have advised waiting until 12 months when post-treatment changes typically settle [30]. Recurrence can be seen as enhancing tissue within an area of scarring.

##### Complications

Overall complications rates are low in FLA [41]. However, the procedure can damage adjacent structures, such as the urethra or neurovasculature. Damage to the rectal wall during fiber positioning may occur if using a transrectal approach. The most commonly reported complication is perineal discomfort. No changes in urinary or sexual functions were reported in Phase I trials [41].

#### Photodynamic therapy

##### Background

PDT was first introduced in the 1990s as a treatment for low- to intermediate-risk prostate cancer using TRUS [43]. Since then, several generations of photosensitizers for PDT have been developed, which are under evaluation [44].

##### Procedure

A 10-min intravenous injection of a photosensitizing agent occurs prior to PDT [45]. This agent is then activated by optical fibers, which are strategically placed with TRUS using MRI fusion guidance [23, 45]. The activation of the photosensitizing agent creates free radicals that target blood vessels and tumor neovasculature, resulting in tissue devascularization. This may have some selectivity for tumor.

##### Post-procedure imaging (Fig. 8)

**Fig. 8:**
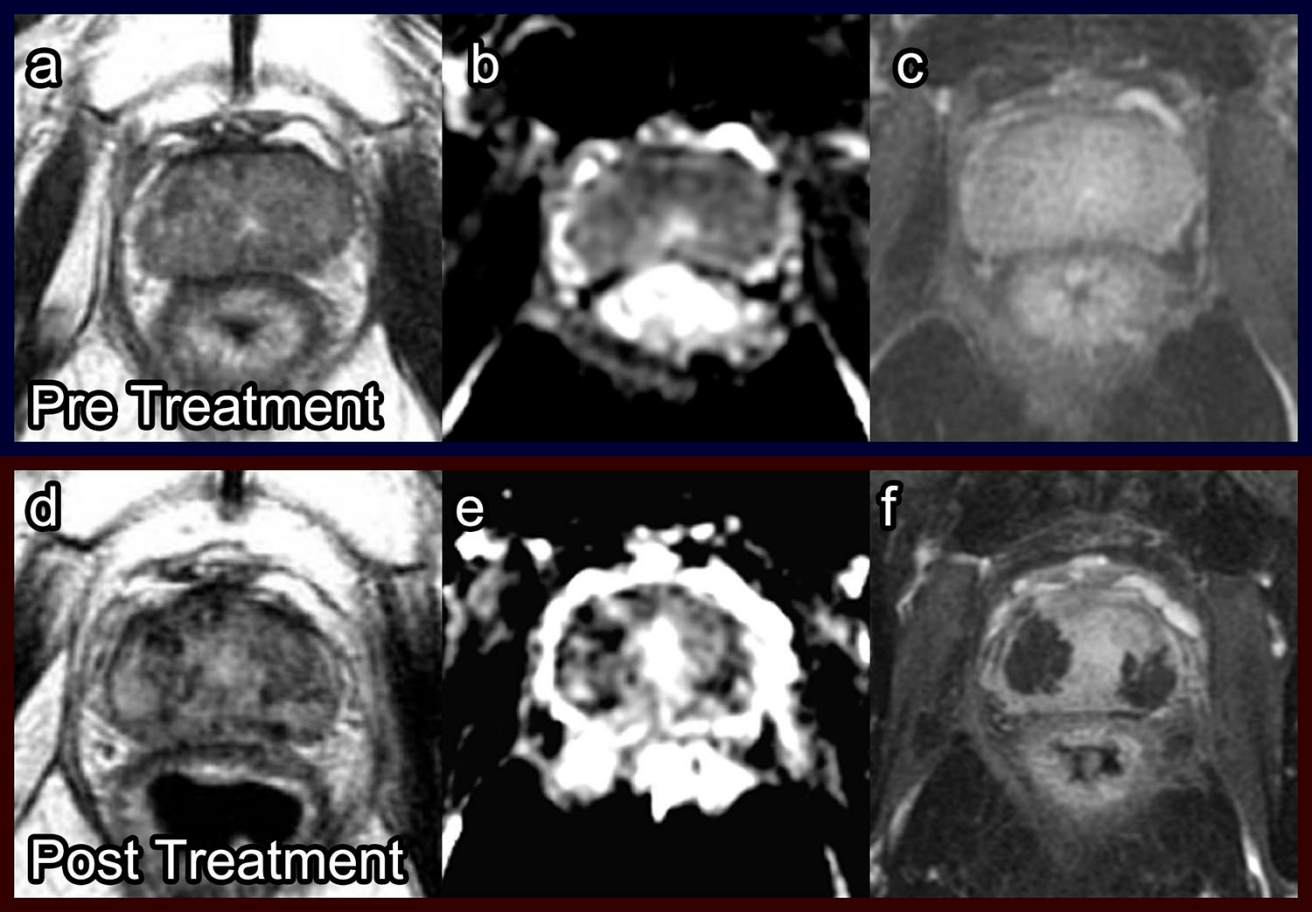
Photodynamic therapy. Pre-treatment prostate MRI in a patient with history of external beam radiation therapy for prostate cancer: T2 (**a**), ADC (**b**), and DCE (**c**) demonstrate heterogeneous prostate enhancement, diffuse restricted diffusion, and heterogeneous T2 dark signal. Post-photodynamic therapy prostate MRI at 7 days: T2 (**d**), ADC (**e**), and DCE (**f**) show post-treatment effect best on the post-contrast imaging with areas of nonenhancement

The amount of tissue edema and necrosis typically peaks around 1 week after PDT [30] and is observed on T1WI as increased signal intensity and on T2WI as the same or decreased signal intensity [23]. T1WI is considered to be more useful than T2WI due to ongoing coagulative necrosis and hemorrhage [30]. Interestingly, because of fiber placement, the borders of the treatment area are often irregular with projections of enhancing viable tissue interspersed with nonenhancing necrotic tissue, which is specific to post-PDT imaging (Fig. 8). Six months after PDT, prostate volume is decreased due to scar tissue formation. Additionally, areas of residual necrosis with fluid can be observed, demonstrating low signal on T1WI and high signal on T2WI (Fig. 8).

**Fig. 9:**
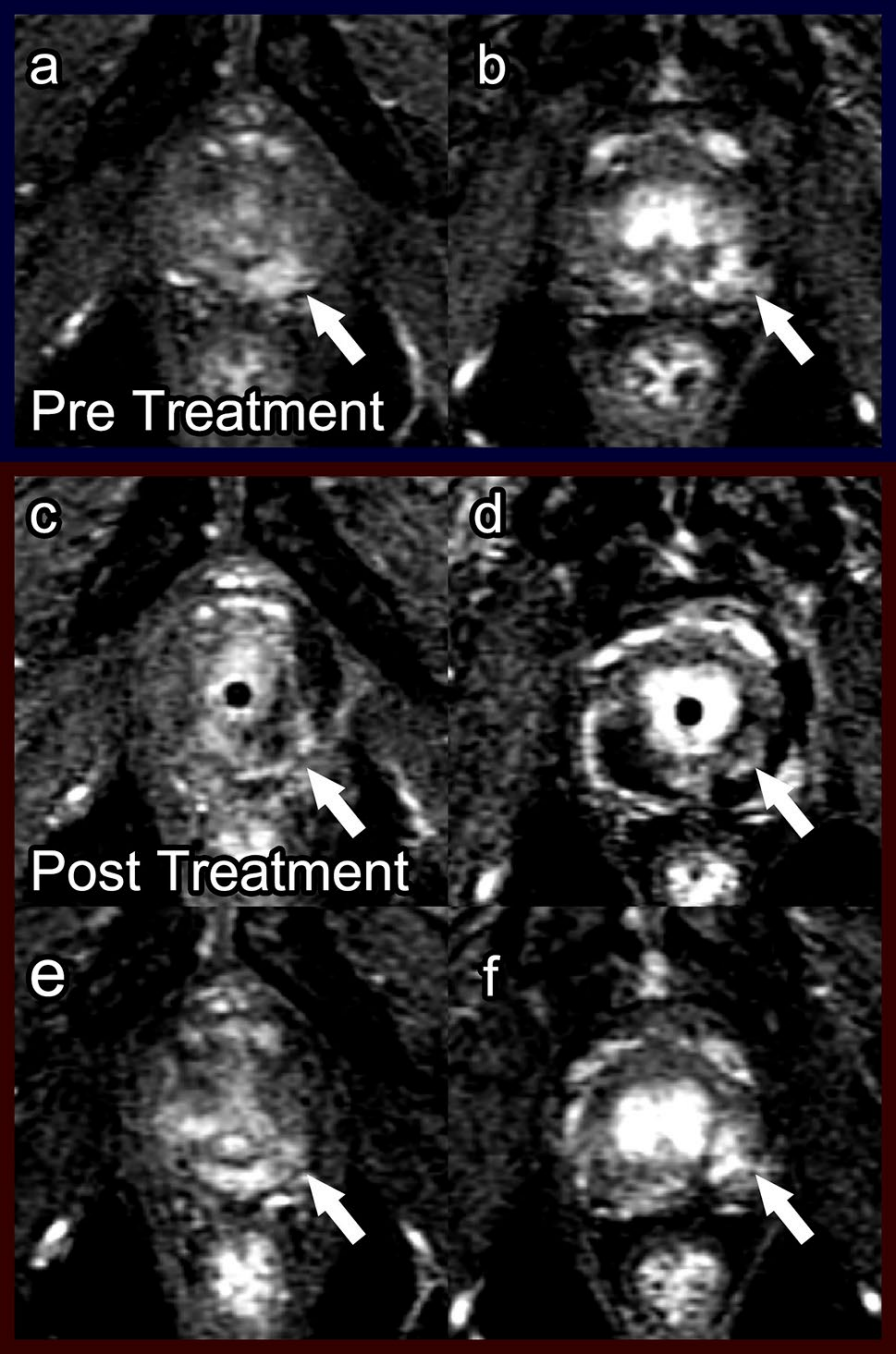
Low-grade recurrence after photodynamic therapy for recurrent prostate cancer after external beam radiation therapy. Pre-treatment prostate MRI: Fat-suppressed DCE during wash-in in the first 30s post-contrast injection (**a**, **b**) showed focal enhancement indicative of tumor in the left mid and apical posterior medial and lateral peripheral zone (arrow). Biopsy in this region showed Gleason score 4 + 3 prostate cancer in 20% and 30% of cores. Bilateral laser fibers were placed in the prostate and activated after injection of WST09 (TOOKAD) as part of a research protocol [23]. Post photodynamic therapy prostate MRI at 7 days: DCE (**c**, **d**) showed bilateral loss of enhancement in the posterior peripheral zone. There were a few tiny spots of residual enhancement (arrow) in the tumor region. Post photodynamic therapy prostate MRI at 6 months: DCE (**e**, **f**) showed increased enhancement in the treated areas (arrow). Biopsy showed low-grade cancer only (Gleason score 3 + 3 in 20% and 5% of the cores) in the treated region

Follow-up mpMRI imaging has been recommended as soon as 1-week post PDT to evaluate treatment success because this coincides with the time period of maximum tissue necrosis [23, 30]. Repeat mpMRI should also be done at 6 months post procedure when damage to nontarget tissue is most apparent to establish a baseline. Detection of tumor recurrence is often difficult to assess on T2WI due to the scar signal. ADC map and DCE sequences are the most effective sequences to evaluate recurrence or residual disease (Fig. 9). Residual tumor displays mild diffusion restriction on the ADC map [46]. Any suspicious nodule with early hyperenhancement on DCE or restricted diffusion on DWI should be biopsied.

##### Complications

**Fig. 10:**
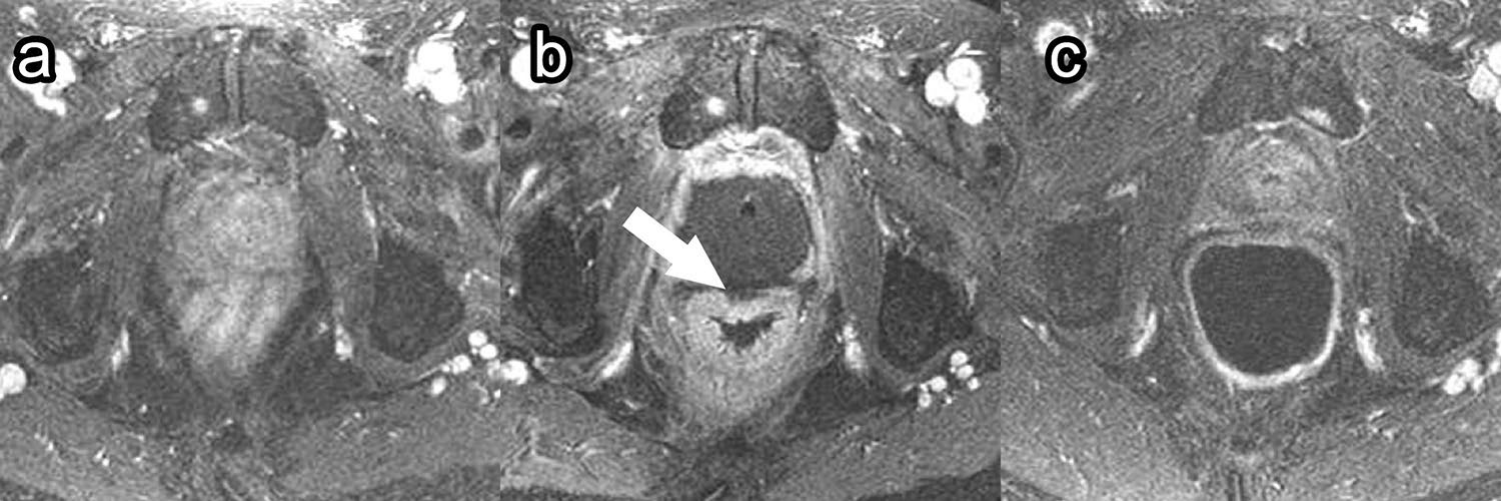
Rectal wall injury following photodynamic therapy. Pre-treatment axial post-contrast T1 (**a**), 1-week post-treatment axial post-contrast T1 (**b**), and 6-month post-treatment axial post-contrast T1 (**c**) images demonstrate focal necrosis to the outer prostate wall (**b**, arrow). At 6-month follow-up (**c**), the rectal wall has healed, and the prostate has decreased in size due to fibrosis

PDT has low overall complication rates [23, 45, 46]. The most common complication of PDT is extraprostatic necrosis, which is generally limited to the periprostatic fatty tissue but can also include the pubic bone marrow, periurethral tissue, rectal wall (Fig. 10), and muscles close to the prostate [23, 46]. This extraprostatic necrosis can be seen as a lack of enhancement on post-contrast sequences. Other common complications include hematuria, urinary urgency, and perineal pain [45].

## Conclusion

Novel minimally invasive procedures can effectively treat BPH and localized prostate cancers. Radiologists reading prostate mpMRI should be familiar with these procedures and the associated complications and normal post-procedure imaging findings to ensure accurate interpretation for evaluation of treatment success and disease recurrence.
